# Reduced Cerebellar Brain Inhibition and Vibrotactile Perception in Response to Mechanical Hand Stimulation at Flutter Frequency

**DOI:** 10.1007/s12311-022-01502-4

**Published:** 2022-12-11

**Authors:** Monica Christova, Victoria Sylwester, Eugen Gallasch, Shane Fresnoza

**Affiliations:** 1 Otto Loewi Research Center, Physiology Section, Medical University of Graz, Neue Stiftingtalstraße 6/D05, 8010 Graz, Austria; 2 Institute of Physiotherapy, University of Applied Sciences FH-Joanneum, Graz, Austria; 3 Institute of Psychology, University of Graz, Graz, Austria; 4 BioTechMed, Graz, Austria

**Keywords:** Cerebellum, Mechanical stimulation, Motor cortex, Sensory cortex, Cerebellar brain inhibition, Vibrotactile perception

## Abstract

The cerebellum is traditionally considered a movement control structure because of its established afferent and efferent anatomical and functional connections with the motor cortex. In the last decade, studies also proposed its involvement in perception, particularly somatosensory acquisition and prediction of the sensory consequences of movement. However, compared to its role in motor control, the cerebellum’s specific role or modulatory influence on other brain areas involved in sensory perception, specifically the primary sensorimotor cortex, is less clear. In the present study, we explored whether peripherally applied vibrotactile stimuli at flutter frequency affect functional cerebello-cortical connections. In 17 healthy volunteers, changes in cerebellar brain inhibition (CBI) and vibration perception threshold (VPT) were measured before and after a 20-min right hand mechanical stimulation at 25 Hz. 5 Hz mechanical stimulation of the right foot served as an active control condition. Performance in a Grooved Pegboard test (GPT) was also measured to assess stimulation’s impact on motor performance. Hand stimulation caused a reduction in CBI (13.16%) and increased VPT but had no specific effect on GPT performance, while foot stimulation had no significant effect on all measures. The result added evidence to the functional connections between the cerebellum and primary motor cortex, as shown by CBI reduction. Meanwhile, the parallel increase in VPT indirectly suggests that the cerebellum influences the processing of vibrotactile stimulus through motor-sensory interactions.

**Supplementary Information:**

The online version contains supplementary material available at 10.1007/s12311-022-01502-4.

## Introduction

Enriched tactile experience conveyed through peripheral mechanoreceptors modulates neuronal excitability not only in the primary somatosensory cortex (S1) but also in the primary motor cortex (M1) because of their reciprocal connections. Peripheral electrical stimulation (e.g., median nerve) modulates neuronal excitability [1–4] increases synaptic density in M1 [5], and increases the somatosensory evoked magnetic field within S1 [6]. Subthreshold random-frequency vibration applied to the wrist, also decreases transcranial magnetic stimulation (TMS)–measured short-interval intracortical inhibition (SICI) and decreased EEG power in the sensorimotor region [7]. Meanwhile, 25 Hz mechanical hand stimulation increases TMS-measured corticospinal excitability and intracortical facilitation (ICF) and reduces SICI [8]. Trains of 22 Hz vibrotactile stimuli applied on the index finger also evoke robust transient onset and steady-state responses within S1 [9], and hand or single-digit mechanical stimulation at flutter frequencies activates S1 and contralateral M1, as well as S2 and posterior parietal cortex in several functional magnetic resonance imaging (fMRI) studies [3, 10–12]. S1 is thought to execute the online processing of somatosensory signals via interactions with M1 during movement [13].

M1 also receives afferent projection from the cerebellum through the cerebello-thalamocortical pathway. Purkinje neurons, the sole output neurons of the cerebellar cortex, have inhibitory connections with the deep cerebellar nuclei, which in turn have a di- or trisynaptic excitatory pathway through the ventral thalamus to M1 layers I, III, V, and VI neurons [14–17]. The cerebellum is implicated not only in various aspects of motor and non-motor learning but is proposed to hold the representation of actions, specifically their temporal encoding, a function crucial for fine-tuning motor output and regulating motor adjustments [18, 19]. Indeed, difficulty producing well-timed movements is a hallmark deficit in patients with cerebellar pathology (e.g., ataxia and dysdiadokinesia). In humans, the phenomenon called cerebellar brain inhibition (CBI) elicited using TMS can be used to prove the cerebellar projection to M1 non-invasively. In CBI, a conditioning magnetic pulse applied to the cerebellum reduces the motor-evoked potential (MEP) amplitude elicited by M1 stimulation 5–8 ms later [20, 21]. TMS of the unilateral cerebellum is thought to inhibit the excitatory dentothalamocortical projection or the pathway from the dentate nucleus to the forelimb zones of M1 [20]. CBI is reduced or absent in patients with a lesion in the cerebellum or cerebello-thalamocortical pathway [22]. In healthy individuals, disrupting cerebellar excitability with an inhibitory repetitive TMS paradigm and cathodal transcranial direct current stimulation (tDCS) decreases CBI [23, 24], while anodal tDCS that increase neuronal excitability enhances it [23]. Moreover, robust CBI modulates intracortical excitability; it reduces SICI and increases ICF [25]. On the other hand, through the cortico-ponto-cerebellar tracts, the cerebellum receives inputs from the contralateral M1, thought to be the efferent copies of a motor command used by the cerebellar cortex to predict the sensory consequences of movements [18].

In humans, a direct anatomical projection from deep cerebellar nuclei to the classic thalamic somatosensory relay nuclei is not yet identified, which according to some assumptions, explains why cerebellar lesions cause uncoordinated movements but not gross sensory deficit [26, 27]. However, animal studies suggest the existence of cerebello-cortical connections. In monkeys, electrically stimulating the anterior interposed nucleus evoked positive BOLD responses in M1 and S1 [28]. Multisynaptic pathways between the cerebellum and S1 were also discovered in rodents [26, 29–31]. Nonetheless, it remains to be explored how cerebellar output modulates activity in S1. On the other hand, descending pathways originating from the motor and sensory cortices, including the cerebro-ponto-cerebellar and cerebro-olivo-cerebellar pathways, terminating as mossy fibers and climbing fibers in the cereberal cortex (respectively), are well documented [29]. It was shown that S1 contributes significantly more corticopontine projections than M1 to the cerebellum [3, 32–34]. Studies suggest that the integration of multimodal inputs from the somatomotor, somatosensory, and other cortices is performed by the cerebellum to exert returning influence on a wide distribution of thalamic and neocortical targets [31]. Magnetoencephalography (MEG) studies in humans show that the cerebellar evaluation of somatosensory input is initiated parallel with cortical processing in S1 [35, 36]. Indeed, disturbances of the contralateral S1 by weak single-pulse TMS or TMS-induced “virtual lesions” delay the implementation of the subsequent load-phase controller during grasping, presumably because of disturbed processing of tactile afferent information and/or sensory predictions [37–39].

The presence of anterograde and retrograde S1-M1-cerebellar connections, the somatotopic representation of the body’s tactile surface in the cerebellum, and rapid direct afferents from sensory structures such as the skin, make the cerebellar circuitry ideally suited for the new proposed role in coordinating the acquisition of sensory data and alerting S1 (and other cortical areas) to these incoming inputs [19, 40–42]. There are studies showing the interplay between the cerebellum, S1, and M1. In conditioning studies in cats, the sequential application of weak electric shock on the interposed nucleus and forelimb skin elicits an increase in M1 excitability [43, 44]. Meanwhile, median nerve stimulation in healthy human subjects evoked stimulus-locked 35–45 Hz oscillations in S1 and 25–35 Hz activity in the cerebellum during the first 100 ms of the processing of the repetitive somatosensory inputs [36]. Other human fMRI and MEG studies also showed the involvement of cerebellar circuits in processing purely sensory information [35, 36, 45, 46]. However, most previous studies explored the effect of somatosensory afferent input on cerebellar’s activity with electrical stimulation. In this regard, the cerebellum’s role in processing more natural sensory inputs, such as vibrotactile stimulation that can extensively activate mechano- and proprioceptors, is not sufficiently understood. At least one study delivered 100 Hz vibratory stimulation on the right hand during finger tapping and showed significant ipsilateral cerebellar BOLD activation [47]. So far, there has been no study on the effect of vibrotactile stimulation at flutter frequencies (20–50 Hz) on cerebello-cortical activity in humans. Therefore, in the present study, we examine whether 25 Hz vibrotactile stimulation modulates CBI, a functional index of cerebellar-M1 connections and vibrotactile perception threshold to tap cerebellar-S1 connections.

## Materials and Methods

### Participants

Seventeen healthy volunteers (9 females, age range = 21 to 34 years, mean age = 25 years, SD = 4.5 years) participated in the study. All had normal or corrected-to-normal vision and were right-handed, according to the Edinburgh Handedness Inventory [48]. None of the participants reported a history of chronic medical or neuropsychiatric disorders (e.g., depression, epilepsy, and stroke), brain injuries, neurosurgical intervention, neuromuscular impairments, intake of medications, substance abuse, pregnancy, and contraindications to TMS such as metallic or electrical implants in the body or the head. The Medical University of Graz Ethics Committee approved the study (ethic number: 10600), and all experimental procedures were conducted following the principles of the Helsinki Declaration regarding human experimentation. Written informed consent was obtained before the experiment. Participants received monetary compensation (35 euros) or course credit points equivalent to the time (3 h) they spent in the study.

### Experimental Design and Procedure

The study was conducted in a single-blinded, sham-controlled design. Each participant took part in two randomized experimental sessions: one session with right hand mechanical stimulation (“HANDSTIM”) and one session with right foot mechanical stimulation (“CONTROL”), which served as an active control/sham condition [8]. The sessions were separated by at least 7 days to avoid carry-over effects. Before the first experiment, each participant was briefed about the purpose of the research and the details of the procedure. After all questions were answered and clarified, baseline measurements of vibration perception threshold (VPT), motor performance (Grooved Pegboard Test or GPT), and CBI (double-pulse TMS) were conducted. Subsequently, the participants underwent either hand or foot mechanical stimulation for 20 min. Repeated measurements of the VPT (immediately after and 30 min after the intervention) and GPT and CBI (immediately after the intervention) were conducted after mechanical stimulation. Overall, including the preparation, one experimental session lasted for 90 min.

### TMS Application and CBI Recording

This part of the experiment started with the localization of the right first dorsal interosseous (FDI) muscle representation in the left M1 using single-pulse TMS (The Magstim Company, Whitland, Dyfed, UK). Participants wore an elastic swimming cap and were seated in a comfortable reclining chair with head and arm supports. They were asked to relax and open their eyes during TMS stimulation. For electromyographic (EMG) recording, Ag–AgCl surface electrodes (9 mm in diameter) were attached to the right FDI muscle in a belly-tendon montage.

The ground electrode was placed on the right wrist. The EMG signals were amplified (1000 ×), bandpass filtered at 8–2000 Hz, digitized at 10 kHz, and recorded using System Plus software (Micromed system, Veneto, Italy) for later analysis. EMG recordings were displayed continuously to ensure the absence of any voluntary motor activity during the TMS assessments. Single magnetic pulses were delivered through a figure-of-eight coil (outer loop diameter of 9 cm) positioned over the left M1 so that the coil intersection was tangential to the scalp (handle pointing backwards and laterally at a 45° angle away from the midline). To locate the M1 site where the largest motor-evoked potential (MEP) can be elicited on the right FDI muscle (FDI “hotspot”), stimulations at suprathreshold intensities were delivered in small steps starting at a point 4 to 5 cm lateral to the vertex. Once located, the stimulation intensity (expressed as a percentage of the maximum stimulator output or MSO) was recorded, and the hotspot was marked on the cap with a colored marker. Subsequently, the resting motor threshold (RMT), defined as the minimum stimulation intensity that can evoke an MEP with approximately 0.05 mV peak-to-peak amplitude in a resting hand muscle, was determined [49].

For CBI measurements, the participants were repositioned with their heads fixed on a metal frame. Initially, the location of the right cerebellar hemisphere was determined and marked on the cap. We followed the procedure by Ugawa et al. and marked a spot 3 cm lateral to the inion on the line joining the inion and the external auditory meatus as the location of the right cerebellar hemisphere [20]. Two monophasic Magstim 200 magnetic stimulators connected via Bistim module (The Magstim Company, Whitland, Dyfed, UK) and two coils (a 110-mm double-cone coil and a 90-mm figure-of-eight flat coil) were used for CBI assessments. A paired-pulse TMS paradigm was delivered to the right cerebellar hemisphere (conditioning stimulus or CS) and the left M1 (test stimulus or TS). The CS was applied using the double-cone coil at an intensity calculated using the individual participant’s shortest scalp-brain distance at the motor cortex (D_M1_) and the cerebellum (D_CEREB_) [50]. These distances were derived from the MRI data of each participant collected from previous experiments they took part (separate study). Individual participants’ data were then entered into the Stoke equation: CS intensity = RMT_M1_ + M (D_CEREB_—D_M1_) [24, 50, 51]. “M” corresponds to the spatial gradient relating RMT to distance, which in the case of unknown, is replaced by an approximate derivation of distance-adjusted MT of a value of 3 [50]. Using the “M” values of 3, the group mean distances estimated from the structural MRI data were D_CEREB_ = 22.15 ± 4.25 mm and D_M1_ = 15.08 ± 1.23 mm. This resulted in cerebellar stimulation intensities between 60 and 75% of the MSO. The current direction induced by the double-cone coil was downward to induce an upward current in the cerebellar cortex [51]. For each participant, the paired-pulse stimulation was started at a CS intensity of 60% and increased in 5% steps until a reliable MEP suppression was observed. On the other hand, the TS was delivered to the left M1 at an intensity that elicited a 1.0 mV peak-to-peak amplitude in the relaxed FDI muscle. The TS followed the CS at a delay of 5 ms, the interstimulus interval known to elicit the best cerebellar inhibitory effect [20, 52]. The ratio of the mean of the “conditioned” MEP amplitude evoked by the CS + TS to the mean of the “unconditioned” MEP amplitude evoked by the TS alone, is the direct measure of CBI. At least twenty conditioned (CS + TS) and 20 unconditioned (TS) MEPs were obtained per time point in a randomized order.

### Vibrotactile Perception Test

The individual participants’ vibrotactile perception threshold (VPT) was measured using the bone vibrator of a clinical Interacoustics-AC33 audiometer (Interacoustics, Denmark). VPT is the lowest amplitude (in decibels) at which vibration can be felt at the pulp of a finger. Participants were first familiarized with the vibration sensation by applying the probe to the hand. For the actual measurements, participants were asked to close their eyes and concentrate. The experimenter placed the vibrator probe on the pulp of the participant’s right thumb. The vibration was turned on at 250 Hz, within the frequency range humans are most responsive to (200–250 Hz) [53]. The vibration was slowly accelerated from 0 to 35 decibels, and the participants were instructed to verbally indicate when they perceived the vibration.

### Grooved Pegboard Test

GPT assesses psychomotor abilities, including fine motor dexterity, motor speed, hand–eye coordination, and executive function [54, 55]. In the present study, performance on the GPT is assessed to explore the effect of mechanical stimulation on motor performance. The pegboard used measures 24 cm × 11 cm and weighs 0.66 kg (Model 32,025, Lafayette Instrument Co. Europe, Loughborough, UK). It contains twenty-five holes arranged in straight lines and is equally spaced (5 × 5 array). The participant’s task is to pick individual pegs or pins with grooves in various orientations from the peg tray using the right thumb and index finger and place them on individual holes (left to right and from top to bottom). A trial starts when the experimenter utters the “ready-steady-go” command and finishes when the participants put the last peg on the last hole. Participants were instructed to insert the pins as fast as possible without missing a hole. The experimenter recorded the time (in seconds) it took for the participants to insert all pegs, which we termed “pegboard completion time.” Each participant was tested with the right hand first, followed by the left hand.

### Mechanical Stimulation

The right hand and foot mechanical stimulation was performed using a Swisswing BMR 2000 device (SwissTTP Inc. USA). This device consists of a 30-cm diameter barrel forced into eccentric motion by an internal electromotor. The noise level generated by the device was low (54 dB). Illustrations can be found in Christova et al. [8]. During the stimulation, the participants sat comfortably on a chair and were instructed to rest their hands (palm side) and foot soles on the barrel. For right hand stimulation (HANDSTIM), the barrel vibrated at a frequency of 25 Hz with a 2 mm peak motion amplitude for 20 min. Mechanical stimulation of the right hand at 25 Hz is known to elicit an increase in corticospinal excitability, a decrease in SICI, and an increase in ICF in the left M1, as well as an increase in blood-oxygenation-level-dependent (BOLD) responses within the left sensory-motor cortex [8, 10]. Meanwhile, for right foot stimulation (CONTROL), the vibration frequency was set to 5 Hz since frequencies below 10 Hz were shown to have no excitatory effects on the left M1 [8]. Another control experiment was conducted on additional 6 participants (2 females, age range = 23–30 years, mean age + SD = 26.50 ± 0.71 years). They underwent the same experimental procedure, albeit received 25 Hz stimulation of the right foot, to exclude the possibility that this stimulation frequency could cause changes in CBI. A two-tailed paired *t*-test revealed that 25 Hz right foot stimulation has no significant effect (*t* = 0.411, *p* = 0.698) on CBI (pre-stimulation: M = 0.71, SE = 0.01; post-stimulation: M = 0.69, SE = 0.02) as shown in Supplementary Fig. 1.

### Statistical Analysis

The raw data were initially inspected offline, and their distribution was tested using the Shapiro–Wilk test. For the CBI data, each trial was checked for involuntary EMG activity preceding the MEP, and their presence justified the omission (none) from the data analyses. The dependent variables CBI (ratio of conditioned MEP/unconditioned MEP), VPT (in dB), and pegboard completion time (in seconds) were then analyzed using three separate repeated measure ANOVAs (SPSS 27.0 software, IBM Corp., Armonk, NY, USA). For the VPT (2 × 3 ANOVA), the independent variables are “stimulation” (HANDSTIM and CONTROL) and time (before, immediately after, and 30 min after mechanical stimulation). For the GPT (2 × 2 × 2 ANOVA), the independent variables are “stimulation” (HANDSTIM and CONTROL), hand (right and left), and time (before and immediately after mechanical stimulation). For CBI (2 × 2 ANOVA), the independent variables are “stimulation” (HANDSTIM and CONTROL) and time (before and immediately after mechanical stimulation). Significant results were explored using post hoc comparisons (paired *t*-test, two-tailed, Bonferroni adjusted for multiple comparisons). Effect sizes are reported as partial eta squared (*η*_*p*_^2^). A *p*-value of < 0.05 was considered significant for all statistical analyses. All values are expressed as the mean ± standard error of the mean (SEM).

## Results

*CBI*: The 17 participants completed the CBI measurements. All participants tolerated the single and double-pulse TMS stimulations well, and there were no reports of headaches, dizziness, or nausea. In the HANDSTIM condition, the mean RMT at M1 was 39.82 ± 0.85% of MSO, and the mean cerebellar CS intensity was 68.94 ± 1.92% of MSO before mechanical stimulation. Meanwhile, the mean RMT at M1 was 39.76 ± 0.88% of MSO, and the mean cerebellar CS intensity was 68.06 ± 1.92% of MSO for the CONTROL condition. A paired *t*-test indicated that the mean stimulation intensity for M1 (*t* = 0.14, *p* = 0.894) and cerebellum (*t* = 1.55, *p* = 0.140) did not significantly differ between stimulation conditions at baseline.

**Fig. 1:**
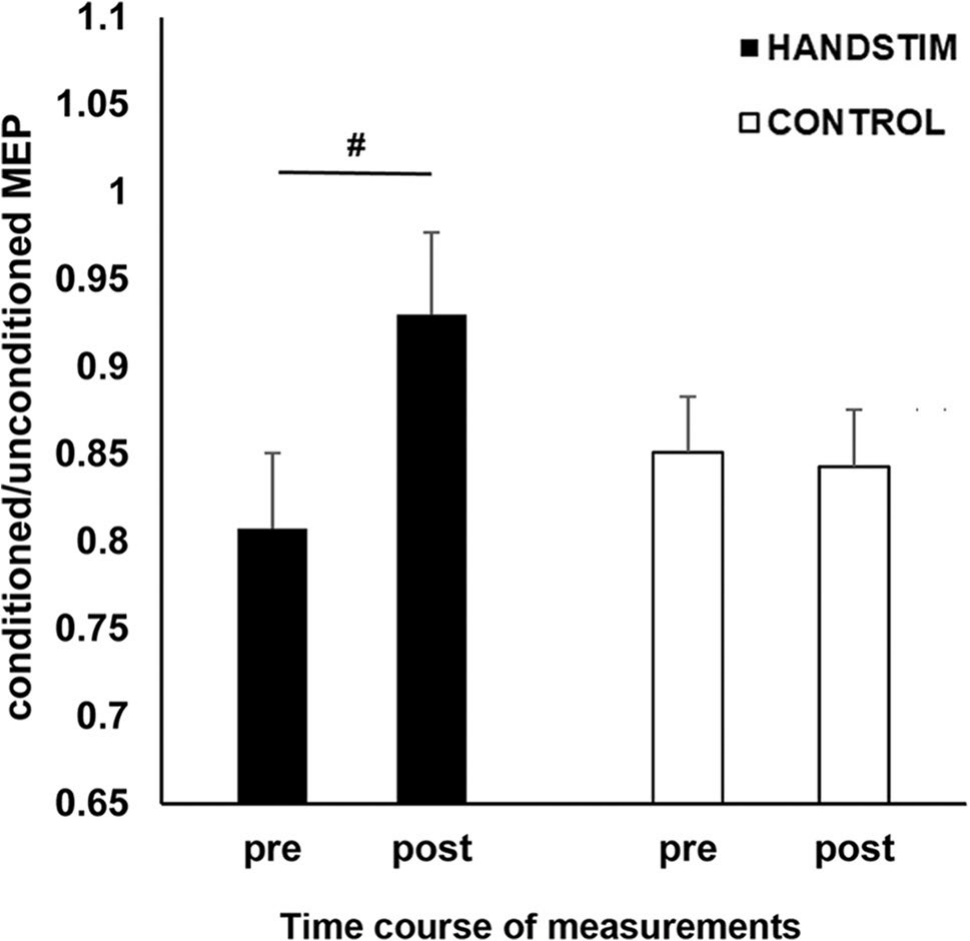
The effects of hand and foot mechanical stimulation on cerebellar brain inhibition (CBI). CBI significantly decrease after mechanical hand stimulation but not after mechanical foot stimulation. The ratios of conditioned/unconditioned MEP data are presented as mean ± SEM. HANDSTIM = mechanical stimulation of the right hand, CONTROL = mechanical stimulation of the right foot, pre = measurement before mechanical stimulation, post = measurements after mechanical stimulation, # = indicate significant differences between the pre and post-stimulation measurements

All data were normally distributed except for the measurement in the control condition before mechanical stimulation (*p* = 0.032). This normality violation was negligible because the skewness (− 0.850) and kurtosis (− 0.478) were smaller than their respective SE (× 3); hence, we proceeded with the parametric testing. The ANOVA revealed a significant main effect of *time* (*F* (1, 16) = 13.10, *p* = 0.002, *η*_*p*_^2^ = 0.450). Mechanical stimulation significantly decreased the overall CBI (before stimulation: M = 0.82, SE = 0.03; after stimulation: M = 0.90, SE = 0.03). The significant *time x stimulation* interactions (*F* (1, 16) = 13.08, *p* = 0.002, *η*_*p*_^2^ = 0.450) indicated the significant reduction in CBI after mechanical hand stimulation (*p* =  < 0.001) (Fig. 1). CBI was comparable before and after mechanical foot stimulation (*p* = 0.911). Bonferroni corrected post hoc test to compare the stimulation conditions in all time points revealed no significant results, consistent with the non-significant main effect of the factor *stimulation* (*F* (1, 16) = 0.04, *p* = 0.836, *η*_*p*_^2^ = 0.003).

Additional paired *t*-tests were performed to explore changes in the MEP responses to the TS and CS + TS. Before mechanical hand stimulation, the mean MEP response to CS + TS (M = 0.95 mV, SE = 0.08 mV) was smaller than response to TS (M = 1.05 mV, SE = 0.07 mV), although the difference was only nearly significant (*p* = 0.094). In contrast, the mean MEP response to the CS + TS (M = 1.00 mV, SE = 0.06 mV) and TS (M = 1.00 mV, SE = 0.09 mV) were equal after hand stimulation (*p* = 0.978). This indicates that the bigger MEP responses to the CS + TS reduce the CBI value after hand stimulation. On the other hand, the mean MEP response to CS + TS (M = 0.92 mV, SE = 0.087 mV) was significantly smaller than the mean response to TS (M = 1.08 mV, SE = 0.07 mV) before mechanical foot stimulation (*p* = 0.003). Similarly, the mean MEP response to CS + TS (M = 0.94 mV, SE = 0.07 mV) was significantly smaller than the mean response to TS (M = 1.11 mV, SE = 0.07 mV) after mechanical foot stimulation (*p* =  < 0.001). These results explain the absence of significant CBI changes in the CONTROL condition.

### *VPT*

**Fig. 2:**
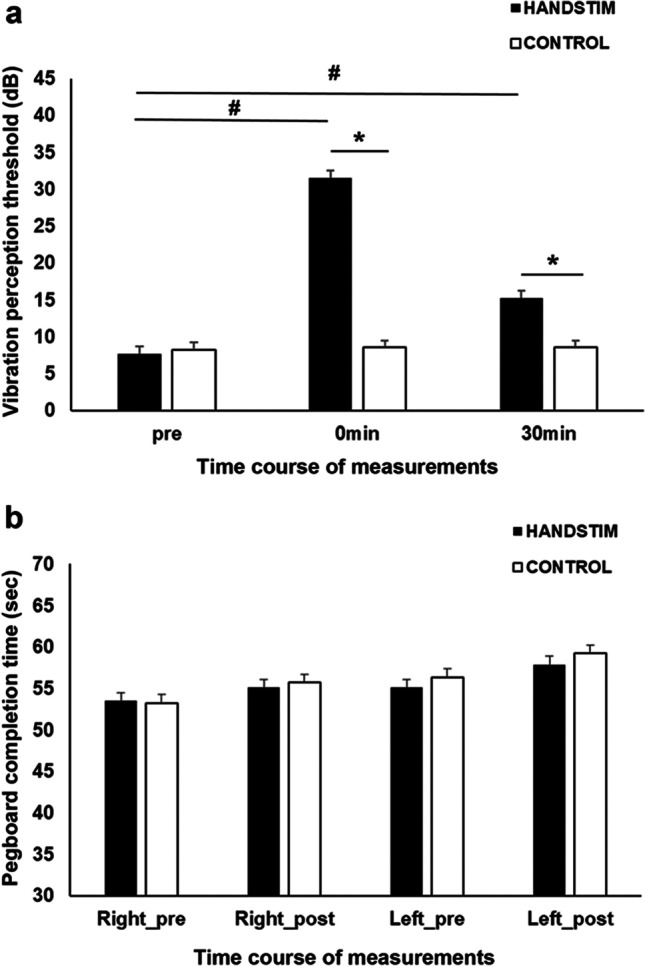
The effects of mechanical stimulation on the control tasks. **a** Vibration perception threshold (VPT) in decibels (dB). VPT significantly increase immediately after and 30 min after mechanical stimulation of the right hand. The increase in VPT was significant compared to the respective time point in the control condition. **b** Pegboard completion time in seconds (sec). There were no significant stimulation-specific effects observed on the completion time. Data are presented as mean ± SEM. HANDSTIM = mechanical stimulation of the right hand, CONTROL = mechanical stimulation of the right foot, pre = measurement before stimulation, right_pre and right_post = measurements conducted on the right and left hand before and after stimulation, left_pre and left_post = measurements conducted on the left hand before and after stimulation, # = indicate significant differences between the pre and post-stimulation measurements, * = indicate significant differences between the HANDSTIM and CONTROL conditions

The 17 participants also completed the vibrotactile perception threshold measurements. The Shapiro–Wilk test indicated that all data were normally distributed except for the pre-intervention measurements in the HANDSTIM condition (*p* = 0.034). Nonetheless, the skewness (0.654) and kurtosis (− 0.546) of this data were negligible (less than 3 × respective SE) and, therefore, would not be problematic for the parametric testing. The results of the ANOVA revealed a significant main effect of *stimulation condition* (*F* (1, 16) = 46.95, *p* =  < 0.001, *η*_*p*_^2^ = 0.746) as indicated by the higher overall VPT in the HANDSTIM condition (M = 18.14 dB, SE = 1.65 dB) than the CONTROL condition (M = 8.43 dB, SE = 1.27 dB) (Fig. 2a). The main effect of *time* was also significant (*F* (2, 32) = 133.90, *p* =  < 0.001, *η*_*p*_^2^= 0.893). Relative to the pre-intervention value, overall VPT significantly increase immediately after (*p* =  < 0.001) and 30 min after (*p* =  < 0.001) mechanical stimulation. The *stimulation and time* interactions was also significant (*F* (2, 32) = 68.17, *p* =  < 0.001, *η*_*p*_^2^ = 0.810). Bonferroni corrected post hoc testing showed that the increase in overall VPT is mainly driven by the significant increase of VPT in the HANDSTIM condition. VPT immediately after (*p* =  < 0.001) and 30 min after (*p* =  < 0.001) hand stimulation were significantly higher than the pre-intervention value (Fig. 2a). Moreover, the VPT immediately after (*p* =  < 0.001) and 30 min after (*p* = 0.001) mechanical hand stimulation were significantly higher than respective values in the control condition.

### *GPT*

The GPT was only performed by the last eight participants because it was added in the middle of the study to serve as an additional control. The data were normally distributed (all *p*s =  > 0.05) based on the Shapiro–Wilk test. The ANOVA performed on the pegboard completion time only showed a significant main effect of *time* (*F* (1, 7) = 11.79, *p* = 0.011, *η*_*p*_^2^ = 0.627). The overall completion time was significantly longer after (M = 57.00 s, SE = 1.86 s) than before (M = 54.56 s, SE = 1.65 s) mechanical stimulation (Fig. 2b). Other main and interaction effects were not significant, as shown in Supplementary Table 1; hence, no further post hoc testing was conducted.

## Discussion

The present study explores whether peripheral vibrotactile stimuli at flutter frequency modulate cerebello-cortical interactions. We measured CBI, VPT, and GPT performance before and after 25 Hz and 5 Hz mechanical stimulation of the right hand and foot, respectively. Hand stimulation significantly reduced CBI and increased VPT but did not affect the GPT performance, while foot stimulation had no significant effect on all measures. The induced reduction in CBI indicates the functional interaction of the motor cortex and the cerebellum in the processing of vibrotactile afferent input.

### Cerebellar Brain Inhibition

The study’s principal finding is the CBI reduction observed as an increase in the amplitude of conditioned MEPs after 25 Hz mechanical hand stimulation. This result may suggest that vibrotactile stimulation reduced the inhibitory output of Purkinje cells in the cerebellar area that receives inputs from the receptive fields in the forelimb skin [56, 57]. Consequently, deep cerebellar nuclei (dentate and interpositus) excitatory output and the ventrolateral nucleus of the thalamus excitatory signals to cortical layers I, III, V, and VI neurons (the deep cerebellar nuclei-thalamocortical pathway) increase [14–17]. The ascending inputs probably originated from the activation of the rapidly adapting type I receptors (RA-I) in the Meissner corpuscles, the most abundant myelinated mechanoreceptors in the human hand (43%), which are sensitive to mid-range (20–50 Hz) frequencies [53, 58–60]. Afferent signals from rapidly adapting type II receptors (RA-II) in Pacinian corpuscles sensitive to high frequencies (60–400 Hz) may also contribute to the somatosensory input through an “assimilation effect.” This occurs when a frequency in the range of one channel influence the perceived frequency on the other channel [53, 59–63]. For instance, Pacinian afferents were reported capable of inducing a vibratory percept at low-frequency spike trains [61]. Co-activation of both mechanoreceptors is also possible through harmonics (e.g., a 30 Hz vibration will generate vibrations of 60, 90, 120 Hz etc.), although at a lower intensity [64]. Moreover, the 25 Hz mechanical foot stimulation does not affect CBI suggesting somatotopic matching between the cerebellar and M1 stimulation sites. However, the question is whether the cerebellum is the source of CBI modulation or does central cortical processing solely mediate the effect?

The cortico-ponto-cerebellar circuits connect S1 and the cerebellum; hence, vibrotactile information relayed by afferent pathways to S1 can modulate CBI [65, 66]. Modulation of cerebellar activity is also possible through M1 projection or the S1-M1 route because of reciprocal connections [67]. Importantly, 25 Hz whole-hand mechanical stimulation induced lasting increase in M1 excitability [8]. We can speculate that the increased neuronal activity in S1 or M1 after mechanical hand stimulation dampened the cerebellar inhibitory output, as indicated by reduced CBI. This is probable since enhancing cortical input by electrical stimulation of a median nerve weakened CBI in humans [68], and direct S1 stimulation modulated the activation timing of inferior olivary neurons resulting in a shortening of climbing fiber response latency in rats [69]. Moreover, increasing corticospinal excitability by delivering trains of repetitive pulses at 10–30 Hz to M1 elicited tremors due to interference of cerebellar afferent inflow to M1 [70].

Cutaneous information from the lower limb through the spinocerebellar tracts (anterior and posterior) and small spino-olivary tract, and the upper limb through the cuneocerebellar and rostral spinocerebellar tracts can also reach the cerebellum [71, 72], and directly modulate CBI. These tracts project into the inferior olivary complex before continuing to the cerebellum as climbing and mossy fibers. They also have exteroceptive components for tactile signals, which in our case, inputs from Meissner and Pacinian mechanoreceptors, as well as proprioceptive components for unconscious information from muscle spindles, Golgi tendon organs, and joint capsules crucial for limb movement coordination and balance [56, 73–75]. The prime position of the inferior olivary complex as a relay node between the spine and cerebellum, as well as being the sole source of climbing fibers, made it a likely structure where the initial influence of afferent input on CBI could occur. The climbing fibers, not mossy fibers, are considered the primary modulator of simple and complex Purkinje cells spikes and therefore govern cerebellar outflow [76]. In addition, because of reciprocal GABAergic connection with deep cerebellar nuclei, the cerebellum regulates the formation of synchronously firing olivary neuron ensembles, which is the proposed mechanism for the “oscillating clock” function of the olivary complex crucial for the appropriate timing of motor command signals [77, 78].

We can speculate that mechanical hand stimulation impairs cerebellar activity because its frequency (25 Hz) does not match the characteristic 1 and 10 Hz resonance frequency of the olivary cells [18, 79]. Downstream effects within the cerebellum could include the reduction of Purkinje neurons’ inhibitory inputs upon cerebellar nuclei, which can cause high discharges of about 40–50 Hz to occur in the latter, particularly in the absence of motion [18, 80], like in our case. Suppose the impact of mechanical hand stimulation on the inferior olivary complex is negligible; there could still be a direct detrimental effect on cerebellar activity because of resonance frequency mismatching. For instance, in rats, granule cell layer neuronal firing oscillates at 7 to 8 Hz, a frequency that correlates with tactile input rather than movement or any movement parameter [81, 82]. The characteristic frequency of simple spikes mediated by the mossy fiber system is also, on average, 30–40 Hz [36]. On the other hand, foot mechanical stimulation may not impair nor affect CBI since its frequency (5 Hz), although within the range of olivary cells’ resonance frequency, is too slow to modulate downstream oscillations.

The impact of vibrotactile stimulation on brain structures located along the intricate dentatothalamocortical pathway, such as the thalamus, might also contribute to reducing CBI. Afferent vibrotactile input can reach the thalamus via two possible routes: the dentatothalamic pathway that terminates on the posterior part of the ventrolateral nucleus before preferentially projecting to M1 and the spinothalamic pathways that terminate in the ventral posterior nucleus, then relay to S1 and M1 [83–85]. The thalamus is an important structure for encoding information about vibrotactile stimuli. Indeed, thalamocortical projection neurons in animals display phase-locked responses to vibration up to 300 Hz [86], while in humans with pathological tremors, neuronal activity in the cerebellum-recipient thalamus also oscillates at or near the tremor frequency [87, 88]. If this is the case, the 25 Hz mechanical hand stimulation can also drive thalamic oscillation, which in our opinion, is disruptive because the dominant oscillation in the cerebello-thalamo-motor cortical-cerebellar loop that regulates discrete micromovements is only between 6 and 10 Hz [85, 89]. Inhibiting the excitatory dentatothalamic projection to M1 will result in bigger conditioned MEP responses and weak CBI.

There is also a strong interaction between the premotor cortex and M1 hand representation [90–92], as well as cerebellar projections to the dorsal and ventral premotor regions [93, 94]. Therefore, it is reasonable to argue that modulation of the premotor cortex could influence the MEP response elicited by paired-pulse stimulation of the cerebellum and M1. This was demonstrated by Spampinato et al. [95] when they found that cerebellar output differentially suppresses MEPs elicited by posterior-to-anterior (PA) currents that directly target the excitability of M1 layer 5 pyramidal neurons (maximal suppression at 5 ms interstimulus interval) and anterior-to-posterior (AP) current that target the excitability of neurons in the premotor cortex that project to M1 (maximum suppression at 7 ms interstimulus interval) [95, 96]. In our study, however, the premotor cortex’s modulation should be minimal since we only used PA currents to stimulate M1 (coil handle points backward at a 45° angle to the midline). On the other hand, regarding the impact of mechanical hand stimulation on the premotor cortex, we proposed two possibilities that may increase MEP responses. First, an increase in M1 neuronal activity might dampen the premotor cortex’s inhibitory output onto it. Second, the inhibition of the premotor cortex following a reduction in dentatothalamocortical output due to cerebellar and thalamic activity disruption [97].

Finally, spinal mechanisms may also influence the CBI magnitude. For example, the H-reflex amplitude, which reflects the excitability of the spinal motoneuron pool, was facilitated by cerebellar TMS, probably through the reticulospinal and rubrospinal tracts [98, 99], as well as by afferent sensory inputs (electrical) during paired associative stimulation [100]. In theory, the increase in spinal motoneuron excitability may counteract the CBI-conditioned descending volleys; hence, the evoked MEPs will be of larger amplitude. However, we can exclude this possibility since 25 Hz hand mechanical stimulation did not induce any spinal effects [8].

### Vibrotactile Perception

The perception of vibrotactile stimuli decreased transiently for 30 min after mechanical hand stimulation, as indicated by the significant increase in VPT. This result concurs with previous reports that exposure to hand-transmitted vibration could produce a temporary or chronic deterioration of finger vibrotactile perception [101–108]. Some studies suggest that the reduction in vibrotactile sensibility is due to hypoperfusion [103, 105, 108, 109]. However, this seems unlikely because the duration of mechanical stimulation is short, and no participants reported numbness or tingling sensation on the right hand. Another possible mechanism would be “afferent-induced inhibition” by tactile inputs, which refers to the elevation of cutaneous vibration detection threshold due to the reduction in neuronal gain (the ratio of the neuron output increment to the input increment) and expansion of the input or stimulus range over which the neuron responds [102]. Animal and human studies suggest that Pacinian corpuscles exclusively or predominantly mediate afferent-induced inhibition at a second-order neuron (cuneate nucleus), which is then distributed on different classes of tactile neurons, including those from the Meissner corpuscle [102, 110–113]. Afferent-induced inhibition is possible at flutter frequency because the RA-II receptors in the Pacinian corpuscles can be activated at low frequencies (e.g., 30 Hz) or through Meissner’s corpuscles, as shown in humans [102]. Afferent-induced inhibition at the cuneate nucleus could also partly explain the absence of VPT modulation after mechanical foot stimulation since somatosensory inputs from the lower limbs converge in another dorsal column nucleus, the gracile nucleus.

A cerebellar-mediated mechanism underlying the impaired efficacy of vibrotactile processing in S1 is challenging to formulate because no disynaptic cerebellar-SI projections have been reported through the cerebellar recipient thalamic relay nuclei in humans [27]. Animals studies, particularly in rats, however, identified thalamic rostral intralaminar nuclei (e.g., central medial and central lateral nucleus) and caudal intralaminar nuclei (e.g., center médian and parafascicular nucleus) that receive inputs from deep cerebellar nuclei (e.g., dentate, fastigial, and posterior interpositus) and sent output to M1 and S1 [114]. The intralaminar nuclei receive monosynaptic input from the mesencephalic reticular formation and, together with midline nuclei, are proposed to provide the necessary arousal of cortical and subcortical regions, allowing faster execution and greater efficiency of cortical processing or lower thresholds for cortical activation by incoming information, such as tactile and nociceptive stimuli [114]. Therefore, we extrapolate that interference of the Purkinje cell’s response by mechanical hand stimulation (as we argued previously) may affect efferent inputs to the intralaminar nuclei and the processing of vibrotactile input in S1, compromising sensory perception. In support of this assumption, the late component of somatosensory evoked potentials (SEPs), considered to represent the initial processing of the somatosensory input in cortical areas, is impaired in patients with unilateral cerebellar damage [27].

An S1-specific mechanism underlying the impaired efficacy of vibrotactile processing is also difficult to rule out since the processing of flutter and vibration inputs, as well as their transient storage, is an established function of S1 [10, 12, 115–117]. During rest or steady contraction, strong alpha (10 Hz) and beta-band (10–30 Hz) oscillations are observed in the sensorimotor region. The beta-band oscillations in S1 synchronized with those in M1, allowing oscillatory sensory reafference to be interpreted in the context of the oscillatory motor command which produced it [118, 119]. In monkeys, the oscillation at 20 Hz but not 10 Hz in M1 and S1 synchronizes with the EMG oscillation in muscles (corticomuscular coherence) [118]. Similar to movements, peripheral nerve stimulation, tactile stimulation, and even motor imagery can evoke event-related desynchronization (ERD) and event-related synchronization (ERS) of the sensorimotor rhythm [120, 121]. ERD is suggested to reflect the downregulation of intracortical inhibition [122], which may account for cortical neurons’ increased excitability during sensory, cognitive, and motor processing [123]. Interestingly, in elderly adults, the reduction of intracortical inhibition and increased excitability of S1 neurons are suggested to underlie the decline in tactile discrimination performance [124, 125]. Here, we argue that a transient decrease in inhibition could cause a reduction in vibrotactile perception due to a decrease in the signal-to-noise ratio within S1. Only the strongest and most consistent signal can be perceived if interference or neural noise is high.

### Grooved Pegboard Test Performance

The analysis of the GPT performance only revealed a general increase in completion time after stimulation. This indicates that hand and foot mechanical stimulation does not affect the GPT performance on either hand. This result is surprising since we expected the performance to be modulated, particularly on the right hand. Our expectation is based on the fact that the flutter-sensitive RA-I afferent’s response to initial contact is the most important for encoding the amount of friction between fingertips and object surfaces [126]. Phase‑appropriate corrective action to update grip‑to‑load force ratios by shifting the object load from the slipping digit to the other digits during frictional slips is also signalled by RA‑I afferents [37, 127]. Little is known about the cerebellum’s role in processing tactile afferent signals during a task that requires grasping, lifting, reaching, and transporting small objects, like the pins in GPT. Nonetheless, fMRI results indicate that both S1 and the cerebellum are engaged in implementing corrective action programs triggered, for instance, by poor object weight prediction [37]. Therefore, we can speculate that suppose there is a reduction of inhibition from Purkinje cells after mechanical hand stimulation; there will be overactivity of cerebellar nuclei, particularly the interpositus nucleus involved in voluntary corrective movements initiated by the feedback of the movement itself and the dentate nucleus, which contributes to the initiation of a movement triggered by stimuli mentally associated with the task [18, 128]. In theory, this could lengthen the completion time since the participant’s movement will be imprecise. The lengthening of completion time can also be due to incoordination (e.g., overshooting) and may reflect disruption of the sensory information (from the medial cerebellum-controlled muscle spindle system) on which the motor system depends [45]. For instance, grasp stability is lost when digit sensibility is impaired since the capacity to adapt the balance between grip and load forces to object surface properties is inefficient [37]. This may result in excessive grip during the precision lift of pins during the task. However, the absence of significant stimulation-specific and hand-specific effects on the completion time in our study disagrees with the scenarios mentioned above but does not completely rule them out.

One possible reason that impacted the GPT result is that this motor task was not highly complicated, easy to learn, and less susceptible to cerebellar interference. Studies have shown that during learning, cerebellar cortex activation progressively decreases while dentate nucleus activation increases, indicating the transfer of plasticity of the motor engram from cortical to deep nuclear zones [19]. In contrast, dentate nucleus activation diminished so the cerebellar motor activation is partly superseded by the striatal one in the overlearning phase [19]. In other words, cerebellar recruitment reduces in simple tasks because sensorimotor maps can be easily acquired, and action execution automated. In this case, sensory feedback mechanisms play a less significant role, and the performance relies more on feedforward control and activity in a network involving the basal ganglia, the supplementary motor area, and the primary motor cortex [120, 129].

Our results contrast with studies using complex tasks such as motor adaptation paradigm with perturbations. Studies reported a negative correlation between CBI reduction and learning of cerebellum-dependent locomotor adaptation tasks and visuomotor adaptation hand tasks with large perturbations [130–133]: greater learning occurred with a greater reduction in CBI, whereas when learning was less, CBI stayed unchanged [130, 132]. The assumption is that the release of CBI following learning reflects the LTD of Purkinje cells [95, 130], a plastic change within the cerebellum crucial in acquiring the internal representation of movement [132]. In our study, the reduction of CBI was not associated with PGT performance improvement.

The discrepancies between the findings from complex tasks and our results can be, in theory, related to the magnitude of cerebellar recruitment. Performance of complex and motor adaptation tasks will robustly engage the cerebellum because they depend heavily on error-driven adaptive learning for continuous recalibration of motor commands to reduce sensory prediction errors [132]. Meanwhile, for easily learned tasks such as PGT, the error-based mechanisms are less involved; hence, the magnitude of cerebellar recruitment will be less compared to that of the complex motor tasks. Lastly, the absence of PGT performance modulation may have been simply due to the small sample size (*n* = 8) underpowered to detect the impact of mechanical stimulation on motor performance. We consider the role of these potential study limitations in interpretation of the results. Future studies involving a bigger sample size might further explore the interactions between CBI and motor performance in simple and complex tasks.

## Conclusions

The present study demonstrates that peripheral vibrotactile stimuli at flutter frequency influence cerebellar-M1 functional connectivity. These results further our understanding of cerebellar cortical dynamics, particularly within the domain of somatosensory processing. The findings may also shed light on the pathophysiology of complex cerebellar diseases and motivates continued explorations of interventions that target cerebellar-mediated impairments, for example, the abnormally strong functional connections between the cerebellum and other brain areas (e.g., frontal gyrus), considered to underlie cognitive impairment in attention deficit hyperactivity disorder.

### Supplementary Information

Below is the link to the electronic supplementary material.

Supplementary file1 (DOCX 125 KB)

Supplementary file2 (DOCX 13 KB)
