# Supplementary material for: Reduced Cerebellar Brain Inhibition and Vibrotactile Perception in Response to Mechanical Hand Stimulation at Flutter Frequency
Source: Cerebellum. 2022 Dec 11;23(1):67–81. doi: 10.1007/s12311-022-01502-4 (PMC10864223; doi:10.1007/s12311-022-01502-4)
Supplement: Supplementary file 1 — Supplementary file1 (DOCX 125 KB) [file 12311_2022_1502_MOESM1_ESM.docx]

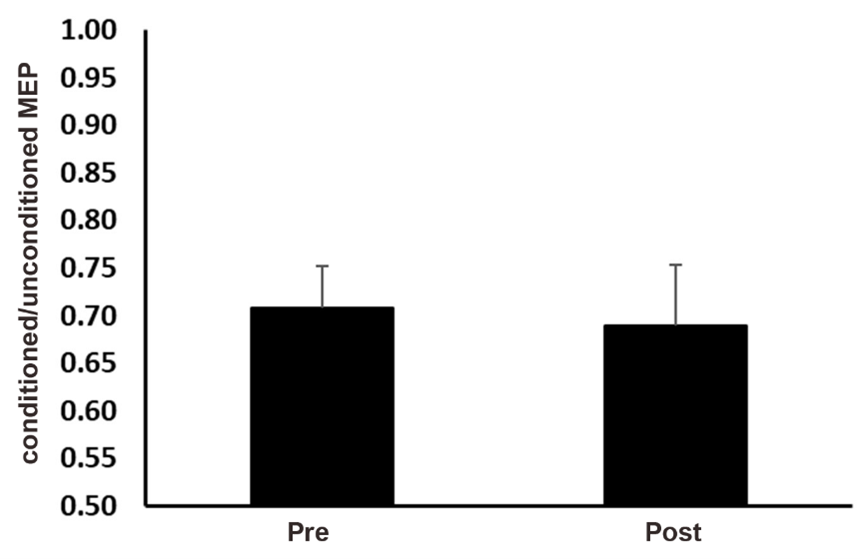


**Supplementary Fig. 1** The effect of 25Hz foot mechanical stimulation on cerebellar brain inhibition (CBI). Bar graphs and vertical error bars depict the mean ± SEM of the CBI ratio. CBI before and after stimulation were comparable (paired t-test, two-tailed). Pre = measurement before stimulation, Post = measurement after stimulation
