# Supplementary material for: Reduced Cerebellar Brain Inhibition and Vibrotactile Perception in Response to Mechanical Hand Stimulation at Flutter Frequency
Source: Cerebellum. 2022 Dec 11;23(1):67–81. doi: 10.1007/s12311-022-01502-4 (PMC10864223; doi:10.1007/s12311-022-01502-4)
Supplement: Supplementary file 2 — Supplementary file2 (DOCX 13 KB) [file 12311_2022_1502_MOESM2_ESM.docx]

**Supplementary Table 1.** Results of the ANOVA performed on the Grooved Pegboard Test completion time before and after mechanical stimulation.

|  | Numerator df | Denominator df | *F*-value | *p-*value | ηₚ² |
| --- | --- | --- | --- | --- | --- |
| Stimulation | 1 | 7 | .25 | .632 | .034 |
| Hand | 1 | 7 | 1.91 | .210 | .214 |
| Time | 1 | 7 | 11.79 | .011* | .627 |
| Stimulation x hand | 1 | 7 | .39 | .551 | .053 |
| Stimulation x time | 1 | 7 | .09 | .769 | .013 |
| Hand x time | 1 | 7 | .11 | .754 | .015 |
| Stimulation x hand x time | 1 | 7 | .11 | .754 | .015 |

Asterisks indicate significant results (*p* < 0.05) df, Degrees of freedom.
